# Molecular Fingerprints of Borderline Changes in Kidney Allografts Are Influenced by Donor Category

**DOI:** 10.3389/fimmu.2020.00423

**Published:** 2020-03-25

**Authors:** Petra Hruba, Zdenek Krejcik, Michaela Dostalova Merkerova, Jiri Klema, Viktor Stranecky, Janka Slatinska, Jana Maluskova, Eva Honsova, Ondrej Viklicky

**Affiliations:** ^1^Transplant Laboratory, Institute for Clinical and Experimental Medicine, Prague, Czechia; ^2^Department of Genomics, Institute of Haematology and Blood Transfusion, Prague, Czechia; ^3^Department of Computer Science, Faculty of Electrical Engineering, Czech Technical University, Prague, Czechia; ^4^Department of Paediatrics and Adolescent Medicine, First Faculty of Medicine, Charles University, Prague, Czechia; ^5^Department of Nephrology, Institute for Clinical and Experimental Medicine, Prague, Czechia; ^6^Department of Pathology, Institute for Clinical and Experimental Medicine, Prague, Czechia

**Keywords:** marginal donor, borderline changes, kidney transplantation, gene expression, microarray

## Abstract

The fate of transplanted kidneys is substantially influenced by graft quality, with transplantation of kidneys from elderly and expanded criteria donors (ECDs) associated with higher occurrence of delayed graft function, rejection, and inferior long-term outcomes. However, little is known about early molecular fingerprints of these events in different donor categories. Borderline changes represent the most frequent histological finding early after kidney transplantation. Therefore, we examined outcomes and transcriptomic profiles of early-case biopsies diagnosed as borderline changes in different donor categories. In this single-center, retrospective, observational study, we compared midterm outcomes of kidney transplant recipients with early borderline changes as a first pathology between ECD (*n* = 109), standard criteria donor (SCDs, *n* = 109), and living donor (LD, *n* = 51) cohorts. Intragraft gene expression profiling by microarray was performed in part of these ECD, SCD, and LD cohorts. Although 5 year graft survival in patients with borderline changes in early-case biopsies was not influenced by donor category (log-rank *P* = 0.293), impaired kidney graft function (estimated glomerular filtration rate by Chronic Kidney Disease Epidemiology Collaboration equation) at M3, 1, 2, and 3 years was observed in the ECD cohort (*P* < 0.001). Graft biopsies from ECD donors had higher vascular intimal fibrosis and arteriolar hyalinosis compared to SCD and LD (*P* < 0.001), suggesting chronic vascular changes. Increased transcripts typical for ECD, as compared to both LD and SCD, showed enrichment of the inflammatory, defense, and wounding responses and the ECM–receptor interaction pathway. Additionally, increased transcripts in ECD vs. LD showed activation of complement and coagulation and cytokine–cytokine receptor pathways along with platelet activation and cell cycle regulation. Comparative gene expression overlaps of ECD, SCD, and LD using Venn diagrams found 64 up- and 16 down-regulated genes in ECD compared to both LD and SCD. Shared increased transcripts in ECD vs. both SCD and LD included thrombospondin-2 (*THBS2*), angiopoietin-like 4 (*ANGPTL4*), collagens (*COL6A3, COL1A1*), chemokine *CCL13*, and interleukin *IL11*, and most significantly, down-regulated transcripts included proline-rich 35 (*PRR35*) and fibroblast growth factor 9. Early borderline changes in ECD kidney transplantation are characterized by increased regulation of inflammation, extracellular matrix remodeling, and acute kidney injury transcripts in comparison with both LD and SCD grafts.

## Introduction

The association of aging with chronic and functional kidney changes has long been acknowledged (1). Kidney recipients from expanded criteria donors (ECDs) are supposed to have inferior midterm renal function and graft survival outcomes (2, 3). In addition to decreasing numbers of functional nephrons, deteriorating alloimmune mechanisms contribute to worse graft outcomes in marginal donors.

Increased transcriptional activation of acute-phase proteins, complement components, and chemokines has been observed during implantation biopsy of grafts from deceased donors vs. living donors (LDs) (4). These underlying molecular mechanisms thus reflect donor organ quality. After transplantation, ischemia/reperfusion injury leads to the up-regulation of inflammation- and apoptosis-related genes due to increased intragraft infiltration of immunocompetent cells (5). This may further aggravate existing injury in ECD grafts.

In recent years, there has been an increase in the use of ECD kidneys [reaching 42–65% (6)] toward meeting demand from patients with end-stage renal disease potentially benefitting from transplantation. Based on midterm follow-up data, marginal donors are associated with inferior renal graft function, higher incidence of delayed graft function (DGF), and infectious complications, despite incidence of acute rejections and long-term graft function being similar to standard criteria donors (SCDs) (2, 7).

In indication biopsies performed early after transplantation, a wide spectrum of diverse diagnoses can be observed, ranging from acute tubular necrosis to T cell– or antibody-mediated rejection. Some of the most frequent findings in early indication biopsies are borderline changes, despite their clinical significance being the subject of debate. Previously, we showed that early borderline changes (BL) biopsies are associated with increased expression patterns of immunity- and inflammation-related genes. Higher donor age as well as some inflammation-related genes additionally contributed to late graft dysfunction (8). Although the transcriptome of kidney graft biopsies in the early period reflects the early alloimmune response, it can also be influenced by ischemia/reperfusion injury and transferred chronic histological changes. While previous study (8) focused on outcomes of BL, in this study on another patient cohort and using different platform, we focus on molecular assessment of various donor categories. The aims of this single-center study were to evaluate renal transcripts associated with donor category in a cohort exhibiting early borderline changes and to identify organ quality-specific patterns, thus limiting any potential bias associated with different histological categories.

## Materials and Methods

### Study Design and Population

**Figure 1:**
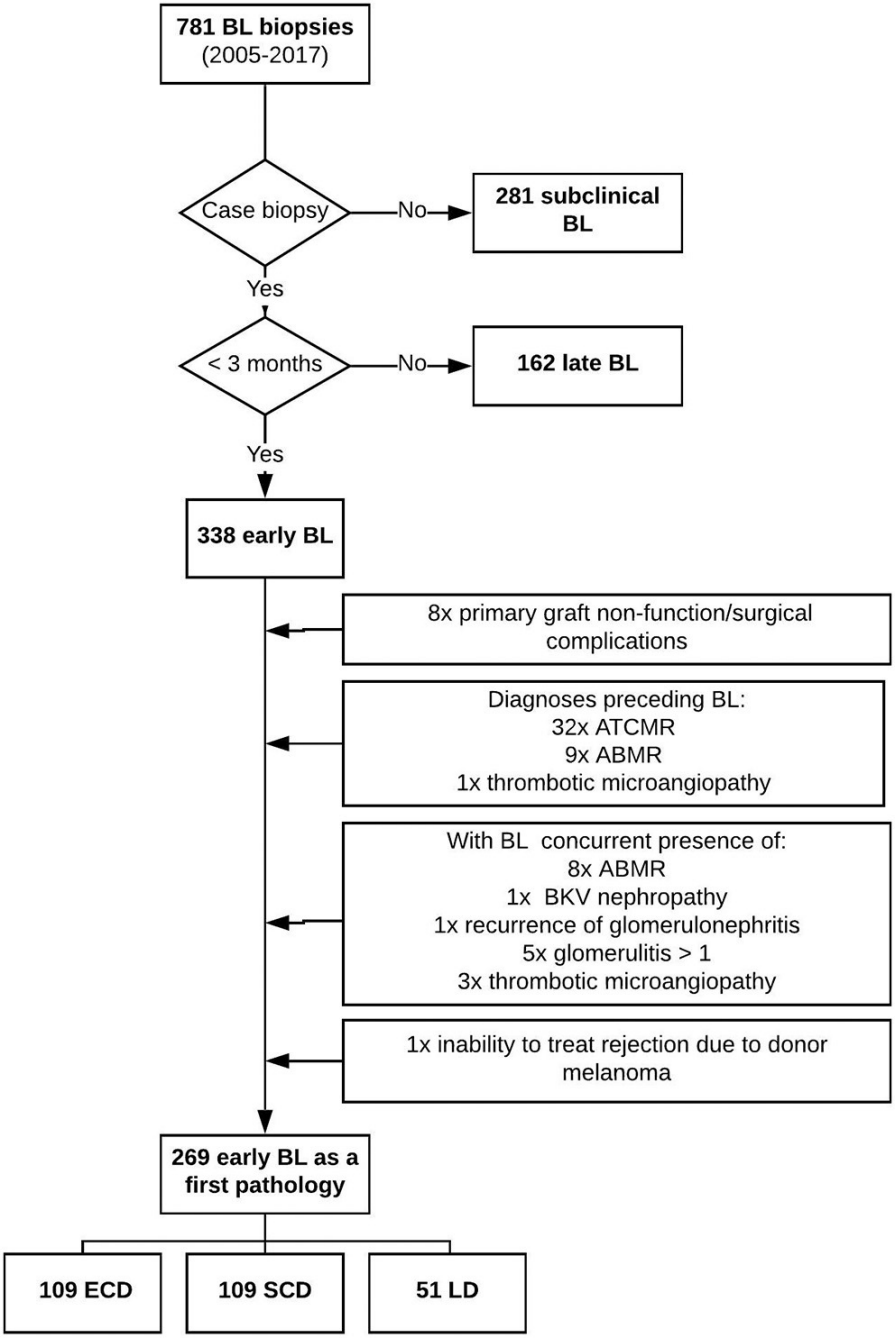
Flowchart of the study.

To study the effect of donor category (deceased vs. living, ECDs vs. SCDs), we carried out a retrospective, single-center, observational, cohort analysis of patients with borderline changes early after transplantation. Of 6,197 kidney recipients transplanted at our center between January 2005 and January 2017, all borderline changes were retrospectively identified (12.6%). Only patients with BL from case biopsies performed early after transplantation [median 9 days (min 4, max 60)] were enrolled in our study cohort (*n* = 338) (Figure 1). To obtain a cohort of borderline changes as a first pathology, all cases with prior episodes of rejection or thrombotic microangiopathy were excluded. To determine pure BL pathology, cases with concurrent presence of antibody-mediated rejection (ABMR), thrombotic microangiopathy (TMA), recurrent glomerulonephritis, glomerulitis >1, and BK virus (BKV) nephropathy were excluded. Furthermore, patients with primary graft dysfunction were deemed ineligible to participate in the study. A final cohort of 269 patients with early BL biopsies as a first and sole pathology was formed, with midterm outcomes compared between ECD (*n* = 109), SCD (*n* = 109), and LD (*n* = 51) categories. Expanded criteria donor kidneys were obtained from deceased donors either aged ≥ 60 years or 50–59 years meeting at least two of the following conditions: serum creatinine >1.5 mg/dL (132.5 μmol/L), cerebrovascular accident as a cause of death, or history of hypertension (9). Standard criteria donors are all deceased donors who failed to meet the criteria for ECD (10). Living donor kidney transplantation was performed between ABO-compatible genetically related or unrelated relatives or friends or with non-directed donors when kidney paired donation was performed. All kidney transplant recipients were treated according to standard center protocol, receiving no induction, T cell–non-depletive (basiliximab, daclizumab) induction, or T cell–depletive induction (rATG or infliximab) followed by a standard triple immunosuppression regimen based on a combination of tacrolimus/cyclosporine, mycophenolate mofetil (MMF)/mycophenolic acid (MPA), and steroids.

For the purpose of the transcriptomic study, we analyzed only patients receiving no induction or non-depletive induction therapy to eliminate the effect of different posttransplant immunosuppression on expression profiles. Furthermore, only biopsies performed within the first 14 days after transplantation were analyzed to reduce time-dependent changes in transcriptional profiles. Thus, the final cohort for molecular analysis consisted of 21 patients across 3 donor categories: ECD, SCD, and LD. Demographics of the microarray cohort are given in Table 1.

**Table 1:** Demographics of patient groups analyzed by microarray.

	**SCD (*n* = 4)**	**ECD (*n* = 9)**	**LD (*n* = 8)**	***P***
Recipient age, years	44 [38, 60]	49 [31, 65]	50 [21, 53]	0.567
Recipient gender, male, *n* (%)	3 (75%)	8 (88.9%)	7 (87.5%)	0.791
Donor age, years	35 [4, 53]	58 [4, 67]	49 [30, 63]	0.044
Donor gender, male, *n* (%)	0 (0%)	5 (55.6%)	2 (25%)	0.119
Dialysis vintage, months	10 [6, 56]	13 [1.6, 20]	7 [0, 31]	0.401
HLA mismatch	3 [3, 4]	4 [1, 5]	5 [2, 5]	0.478
Peak PRA	1 [0, 4]	2 [0, 12]	0 [0, 3]	0.144
DGF, *n* (%)	0	2 (22.2%)	1 (12.5%)	0.563
Cold ischemia, h	17 [9, 18]	17 [11, 22]	0.7 [0, 1.5]	0.001
Induction treatment				0.037
No	0	4 (44.4%)	0	
Basiliximab	4 (100%)	5 (55.6%)	8 (100%)	
Creatinine at biopsy, μmol/L	179 [169, 213]	397 [175, 651]	185 [126, 486]	0.016
Biopsy post-operative day (POD), days	8 [6, 13]	10 [6, 12]	6.5 [5, 13]	0.432

The study was approved by ethics committee of the Institute for Clinical and Experimental Medicine and Thomayer Hospital With Multi-center Competence under number G-16-06-09.

### Microarray Analysis

Total RNA was isolated from renal biopsies using the RNeasy Micro Kit (Qiagen, Hilden, Germany). RNA quality and integrity were determined using the Agilent RNA 6000 Nano Kit on the Agilent 2100 Bioanalyzer (Agilent Technologies, Santa Clara, CA, USA). Samples with an RNA integrity number of <6 were excluded from the analysis. RNA concentration was determined using a Qubit® fluorometer with Qubit® RNA BR Assay (ThermoFisher Scientific, Waltham, MA, USA).

A total of 150 ng RNA served as a template for the amplification and generation of Cy3 fluorescent cRNA using the Low Input Quick Amp Labeling Kit, one-color (Agilent Technologies), according to the manufacturer's instructions. Labeling efficiency, yield, and purity of cRNA were determined using a NanoDrop spectrophotometer. Labeled cRNA (700 ng with specific activity >10.0 pmol Cy3/μg cRNA) was hybridized to Agilent SurePrintG3 Human Gene Expression v3 8×60K Array at 65°C for 17 h in a rotating hybridization oven at a speed of 10 rounds per minute. After hybridization, microarrays were washed sequentially for 1 min in wash buffer 1, for 1 min with prewarmed (37°C) wash buffer 2 (Agilent Technologies) and then immediately dried and scanned. Scanning was performed on the Agilent C Microarray Scanner, with data extraction and quality control performed using Agilent Feature Extraction Software (version 10.7.3.1). The resulting text files were analyzed using R software. The R software Lumi package was used to process raw data obtained from microarray analysis, with the quantile method used for normalization. Raw data sets used in the study were deposited at the Gene Expression Omnibus database (11) under ID GSE134386. When comparing particular donor subgroups, only two genes, *PRR35* and *CD163L1*, differentially expressed between ECD and LD, remained significant after multiple corrections [(false discovery rate (FDR) *P* < 0.05, fold change >2]. Therefore, in further analysis, differentially expressed genes were chosen as those with a fold change >2 and an unadjusted *P* < 0.05. Affected genes were functionally annotated, with deregulated pathways identified using the David database (http://david.abcc.ncifcrf.gov). In order to compare lists of deregulated genes, we availed of an interactive online tool for Venn diagrams (http://bioinfogp.cnb.csic.es/tools/venny/index.html).

### Statistics

Data normality was tested using the Kolmogorov–Smirnov test. As most variables exhibited non-normal distribution, we compared two groups using the two-tailed Mann-Whitney *U*-test and three groups using the Kruskal–Wallis test, followed by the *post hoc* Dunn multiple-comparisons test. Categorical data were compared using the χ^2^ or Fisher exact test. Differences in kidney graft function between SCD, ECD, and LD were calculated using the General Linear Model (GLM) repeated-measures model. Graft survival was compared using Kaplan–Meier estimates and the log-rank test. Two-sided *P* ≤ 0.05 was considered statistically significant.

## Results

### Effect of Donor Category on Graft Outcomes

We compared midterm outcomes of kidney transplant recipients with borderline changes as a first pathology diagnosed at a median of 9 days after transplantation between ECD (*n* = 109), SCD (*n* = 109), and LD (*n* = 51) kidney transplantation cohorts. The ECD group had not only older donors but also higher recipients age (*P* = 0.029) and longer cold ischemia times than did the SCD group (*P* = 0.020). The LD group had the lowest recipient ages, cold ischemia times, and panel-reactive antibody levels and also the shortest dialysis spans (*P* < 0.001). The LD group contained a significantly higher proportion of female donors (*P* < 0.001) (Supplemental Table 1). The highest incidence of DGF was in the ECD group (37%) followed by SCD (32%), with prevalence of DGF only 6% in the LD group (*P* < 0.001).

The effect of donor category on individual Banff indication biopsy scores with borderline change findings showed the greatest chronic changes in the ECD group (Table 2). Graft biopsies from ECD donors revealed significantly higher vascular intimal fibrosis (cv) and arteriolar hyalinosis (ah) compared to both SCD and LD (*P* < 0.001), pointing to chronic vascular changes as well as higher tubular atrophy scores (ct) in grafts from marginal donors. On the contrary, biopsies from the LD group had significantly lower arteriolar hyaline thickening (aah) scores than those from the ECD group (*P* < 0.001) and SCD group (*P* < 0.05).

**Table 2:** Histological findings in indication biopsies with BL performed early after transplantation stratified according to donor type.

**Banff score**	**SCD (*n* = 109)**	**ECD (*n* = 109)**	**LD (*n* = 51)**	***P* (ANOVA)**
Glomerulitis (g)	0.12 ± 0.33	0.11 ± 0.31	0.17 ± 0.4	0.592
Chronic glomerulopathy (cg)	0.01 ± 0.1	0 ± 0	0 ± 0	0.482
Interstitial inflammation (i)	0.52 ± 0.53	0.49 ± 0.54	0.45 ± 0.58	0.754
Tubulitis (t)	1.22 ± 0.59	1.23 ± 0.61	1.22 ± 0.61	0.989
Total inflammation (ti)	0.51 ± 0.6^1^	0.49 ± 0.57	0.31 ± 0.55	0.121
Tubular atrophy (ct/TA)	0.72 ± 0.54^1^	0.86 ± 0.54	0.67 ± 0.52	**0.047**
Interstitial fibrosis (ci/IF)	0.51 ± 0.59^1^	0.54 ± 0.63	0.45 ± 0.54	0.669
Vascular intimal fibrosis (cv)	0.83 ± 0.76^5^	1.34 ± 0.85	0.95 ± 0.72	**<0.001**^**a, d**^
Arteriolar hyalinosis (ah)	0.98 ± 0.75	1.35 ± 0.78	0.82 ± 0.72	**<0.001**^**b, c**^
Arteriolar hyaline thickening (aah)	0.35 ± 0.65	0.39 ± 0.75	0.14 ± 0.40	0.061^c, e^
Peritubular capillaritis (ptc)	0.11 ± 0.44^2^	0.09 ± 0.35	0.06 ± 0.31	0.713

*Dunnett post hoc test confirmed significant differences between SCD and ECD at ^a^P < 0.001 or ^b^P < 0.01; ECD and LD at ^c^P < 0.001 and ^d^P < 0.05; SCD and LD at ^e^P < 0.05. Significant p values are in bold. The numbers in superscript indicate missing data. ANOVA, analysis of variance*.

Patients from the ECD group had significantly worse renal graft function at biopsy, at 3 months, and in the first, second, and third years after biopsy [medians of estimated glomerular filtration rate (eGFR): 0.29, 0.59, 0.64, 0.67, 0.65 mL/s] compared to patients in the SCD (medians of eGFR 0.39, 0.79, 0.83, 0.84, 0.85 mL/s) and LD groups (medians of eGFR: 0.55, 0.79, 0.87, 0.85, 0.94 mL/s) (*P* < 0.001). The renal function of patients who received grafts from LD was better at biopsy compared to the SCD group (*P* < 0.001), despite no differences being found thereafter (Supplemental Figure 1).

Neither 5 year graft survival nor rejection-free intervals significantly differed among recipients with early borderline changes based on donor category (log-rank *P* = 0.293 and 0.219, respectively) (Supplemental Figure 2).

### Effect of Donor Category on the Intragraft Transcriptional Profile of Early Borderline Changes

The effect of donor category on the intragraft transcriptional profile was studied in sections of the ECD (*n* = 9), SCD (*n* = 4), and LD (*n* = 8) cohorts. All biopsies were clinically indicated at a median of 9 days after transplantation (min 5, max 13 days) and diagnosed as borderline changes. There was no difference in the follow-up to biopsy among the ECD, SCD, and LD cohorts (*P* = 0.432). All patients had received their first transplants, had low levels of panel-reactive antibodies, and therefore received no induction or basiliximab. The demographics of this microarray set of patients are given in Table 1. Differences between groups, such as older donors (*P* = 0.044) in the ECD group or shorter cold ischemia times (*P* = 0.001) in the LD group, reflect particular donor category definitions. In addition, patients from the ECD group had the worst renal function at biopsy (median of creatinine was 397 μmol/L for the ECD group, 177 μmol/L for the SCD group, and 185 μmol/L for the LD group, *P* = 0.016).

Similar to our analysis of the larger clinical cohort (Table 2) also in microarray-analyzed biopsies, patients from the ECD group had significantly higher vascular intimal fibrosis (cv) (*P* = 0.028, Supplemental Table 2).

In the ECD group, microarray revealed higher expression of 244 transcripts compared to SCD and 437 compared to LD. Compared to both SCD and LD, gene annotation analysis of transcripts with increased expression in ECD grafts showed enrichment of the inflammatory response (*P* = 0.013, *P* = 7.4 × 10^−8^, respectively), the response to wounding (*P* = 0.001, 1.3 × 10^−12^, respectively), the defense response (*P* = 0.005, *P* = 5.5 × 10^−7^, respectively), and the ECM–receptor interaction pathway (*P* = 0.043, *P* = 0.004, respectively) (Table 3). Additionally, annotation analysis of increased transcripts in ECD vs. LD showed activation of complement and coagulation cascades (*P* = 0.0039), cytokine–cytokine receptor interaction pathways (*P* = 0.02), and other Gene Ontology (GO) terms such as regulation of the cell cycle process (*P* = 1.9 × 10^−6^) and platelet activation (*P* = 0.0001) (Table 3). Interestingly, GO term response to wounding was more activated in ECD kidneys in comparison with SCD (*P* = 0.001) and LD kidneys (1.3 × 10^−12^), and similarly, it was higher in SCD kidneys compared with LD ones (*P* = 0.005). Activation of the KEGG complement and coagulation cascades pathway was observed in both deceased donor categories (ECD and SCD) in comparison with LD (*P* = 0.039 and *P* = 0.019, respectively). Moreover, higher regulation of lipid transport was observed in SCD vs. LD (Table 3).

**Table 3:** Biological processes and KEGG pathway–enriched case biopsies with borderline changes in ECD compared to SCD, in ECD compared to LD, and in SCD compared to LD.

**Enriched biological processes and KEGG pathways**	**Transcripts increased in ECD vs. LD**	**Fold enrichment**	**Benjamini *P***
hsa04610: complement and coagulation cascades	*F12, FGG, FGA, C3, CFB, FGB, F13A1, CFH*	7.37	0.00390
hsa04512: ECM–receptor interaction	*COL6A3, COL3A1, ITGB6, LAMC2, SV2B, ITGB3, COL1A1, THBS2, SPP1*	6.81	0.00370
hsa04060: cytokine–cytokine receptor interaction	*LIF, INHBB, INHBA, TNFSF10, CCL13, IL6, IL2RA, OSMR, CXCL2, TNFRSF18, CCL18, IL11, CCL17*	3.15	0.01960
GO: platelet activation	*FGG, IL6, SAA2, FGA, SAA1, FGB, COL3A1, IL11*	18.28	0.00010
GO: regulation of nuclear division, regulation of mitosis	*NEK2, DLGAP5, BUB1, CENPF, IGF1, CENPE, CDC25C, CD28*	10.45	0.00100
GO: regulation of inflammatory response	*FCER1A, F12, IL6, IL2RA, SAA2, SERPINF1, C3, OSMR, SAA1*	8.66	0.00100
GO: regulation of cell cycle process	*NEK2, DLGAP5, CENPF, IGF1, CENPE, ANLN, BIRC5, UBE2C, CDC25C, GTSE1, LIF, BUB1, CD28*	8.34	0.00000
GO: coagulation, blood coagulation	*F12, FGG, IL6, SAA2, FGA, SAA1, FGB, F13A1, COL3A1, ITGB3, IL11*	7.89	0.00020
GO: acute inflammatory response	*F12, IL6, SAA2, C3, SAA1, CFB, CLU, CFH, SERPINA3, CD163*	7.46	0.00090
GO: hemostasis	*F12, FGG, IL6, SAA2, FGA, SAA1, FGB, F13A1, COL3A1, ITGB3, IL11*	7.45	0.00030
GO: regulation of mitotic cell cycle	*NEK2, DLGAP5, CENPF, IGF1, CENPE, ANLN, BIRC5, UBE2C, CDC25C, GTSE1, BUB1, MYC, CD28*	6.25	0.00020
GO: regulation of body fluid levels	*F12, FGG, IL6, SAA2, FGA, SAA1, FGB, F13A1, COL3A1, ITGB3, AGR2, IL11*	6.22	0.00050
GO: wound healing	*F12, IL6, F13A1, COL3A1, IGF1, ITGB3, CDH3, IL11, FGG, FGA, SAA2, FGB, SAA1, HMOX1, TM4SF4*	5.74	0.00010
GO: inflammatory response	*NFKBIZ, F12, IL6, IL2RA, ELF3, CFB, C3, CLU, CXCL2, GAL, CCL18, CCL17, CD163, FOS, CCL13, SAA2, SAA1, STAB1, HMOX1, ITGB6, SERPINA3, CFH, PTX3, SPP1*	5.40	0.00000
GO: response to wounding	*NRP1, ELF3, C3, F13A1, CLU, CXCL2, COL3A1, ITGB3, CDH3, IL11, FOS, FGG, SAA2, FGA, FGB, SAA1, HMOX1, ITGB6, CFH, SERPINA3, PTX3, SPP1, F12, NFKBIZ, IL6, IL2RA, CFB, IGF1, GAL, CCL18, CD163, CCL17, CCL13, LYVE1, STAB1, VCAN, TM4SF4*	5.10	0.00000
GO: defense response	*ELF3, C3, CLU, CXCL2, HP, FOS, SAA2, SAA1, HMOX1, ITGB6, CFH, SERPINA3, LTF, PTX3, SPP1, F12, NFKBIZ, IL6, IL2RA, CFB, GAL, CCL18, HPR, CD163, CCL17, INHBB, INHBA, CCL13, CD19, LILRB5, STAB1, CTSG*	3.69	0.00000
GO: cell adhesion, biological adhesion	*OLFM4, NRP1, MYBPC2, NELL2, COL3A1, POSTN, ITGB3, SOX9, CDH3, CDH6, VCAM1, COL7A1, COL6A3, ITGB6, SPON2, LOXL2, THBS2, SPP1, COL15A1, CDHR4, LYVE1, STAB1, CD209, CPXM1, CLDN1, VCAN, LAMC2, ADAM12, HABP2*	3.03	0.00010
GO: immune response	*C3, CLU, CXCL2, LIF, CFH, LTF, SPON2, PTX3, CD28, F12, TCF7, IL6, IL2RA, CFB, FOXJ1, RELB, IGJ, CCL18, CCL17, CCL13, TNFSF10, LILRB5, FCGR2B, CD209, CTSC, CTSG*	2.76	0.00090
	**Transcripts increased in ECD vs. SCD**	**Fold enrichment**	**Benjamini** ***P***
hsa04512: ECM–receptor interaction	*TNC, COMP, COL6A3, SV2B, COL1A1, THBS2*	8.30	0.04300
GO: inflammatory response	*CCL11, C1QB, FOS, CCL13, HIF1A, CEBPB, ADORA3, CCL8, C1S, GPR68, GAL, VSIG4, CHST1*	4.40	0.01300
GO: response to wounding	*CEBPB, ADORA3, TNC, CCL8, GPR68, C1S, GAL, IL11, PLAUR, CHST1, CCL11, PCSK1, FOS, C1QB, CCL13, SLC1A3, HIF1A, SERPINE1, VSIG4*	3.60	0.00100
GO: defense response	*ADORA3, CEBPB, KLRC3, CCL8, CD300C, COLEC12, GPR68, C1S, GAL, CHST1, CCL11, INHBA, FOS, C1QB, CCL13, HIF1A, LILRB5, TFF3, VSIG4*	3.40	0.00500
GO: cell adhesion	*TNC, EMILIN2, SIGLEC14, COL16A1, CLDN14, ITGBL1, CCL11, NLGN4Y, COMP, CD33, SIGLEC7, COL6A3, MFAP4, ADAM12, THBS2, COL8A2, NTM, CDH11, SPON1*	3.00	0.01300
GO: biological adhesion	*TNC, EMILIN2, SIGLEC14, COL16A1, CLDN14, ITGBL1, CCL11, NLGN4Y, COMP, CD33, SIGLEC7, COL6A3, MFAP4, ADAM12, THBS2, COL8A2, NTM, CDH11, SPON1*	3.00	0.01100
	**Transcripts increased in SCD vs. LD**	**Fold enrichment**	**Benjamini** ***P***
hsa02010: ABC transporters	*ABCA8, ABCB1, CFTR, ABCB4*	17.12	0.03431
hsa04610: complement and coagulation cascades	*C8A, F12, FGG, CR2, F13A1*	13.65	0.01873
GO: regulation of lipid transport	*APOA2, APOA1, APOC3, PON1*	32.79	0.04672
GO: mitosis, nuclear division	*CCNB2, DLGAP5, CENPF, BIRC5, PBK, UBE2C, ASPM*	7.82	0.0386
GO: response to wounding	*C8A, F12, APOA2, FGG, CR2, F13A1, ITGB3, IGFBP1, CDH3, ADORA1, ORM2, SPP1*	5.57	0.00526

*Only the most significant GO terms associated with biological processes are shown*.

**Figure 2:**
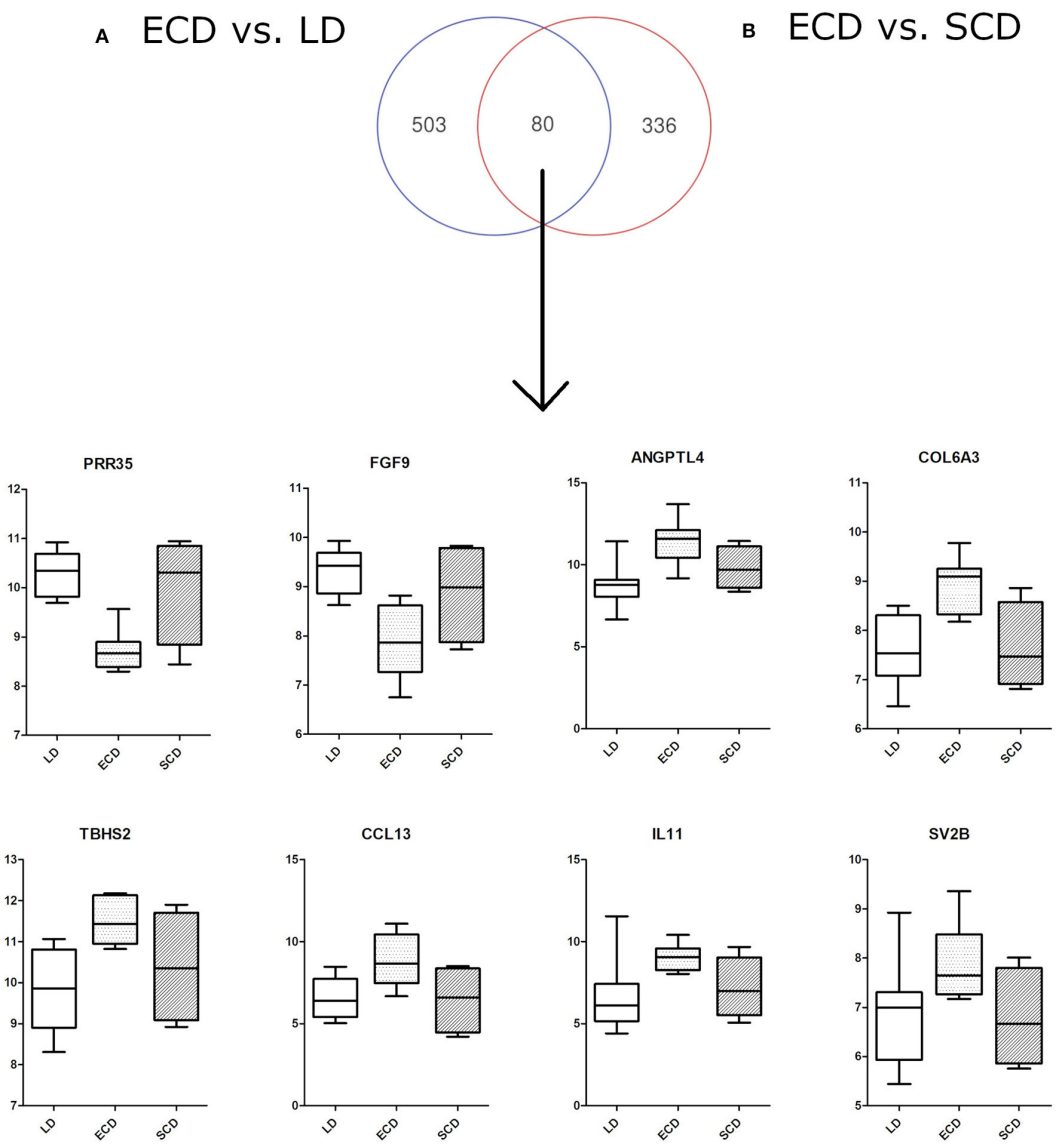
Venn diagram showing overlap of deregulated genes (80 transcripts) for particular comparisons of donor categories: **(A)** ECD vs. LD (583 deregulated transcripts), **(B)** ECD vs. SCD (416 deregulated transcripts). The overlap of some genes shows differential expression of ECD compared with SCD and LD. PRR35, proline-rich 35 protein; FGF9, fibroblast growth factor 9; ANGPTL4, angiopoietin-like 4; COL6A3, collagen, type VI, alpha 3; TBHS2, thrombospondin 2; CCL13, chemokine (C-C motif) ligand 13; IL11, interleukin 11; SV2B, synaptic vesicle glycoprotein 2B.

Comparative gene expression overlaps of differentially expressed genes between ECD vs. SCD and ECD vs. LD using Venn diagrams (Figure 2) found 64 up- and 16 down-regulated genes in ECD compared to both LD and SCD. Shared increased transcripts in ECD vs. both SCD and LD included thrombospondin 2 (*THBS2*), synaptic vesicle glycoprotein (*SV2B*), angiopoietin-like 4 (*ANGPTL4*), collagens (*COL6A3, COL1A1*), chemokines *CCL13*, and interleukin *IL11* and, most significantly, down-regulated transcripts including proline-rich 35 (*PRR35*) and fibroblast growth factor 9 (*FGF9*).

**Figure 3:**
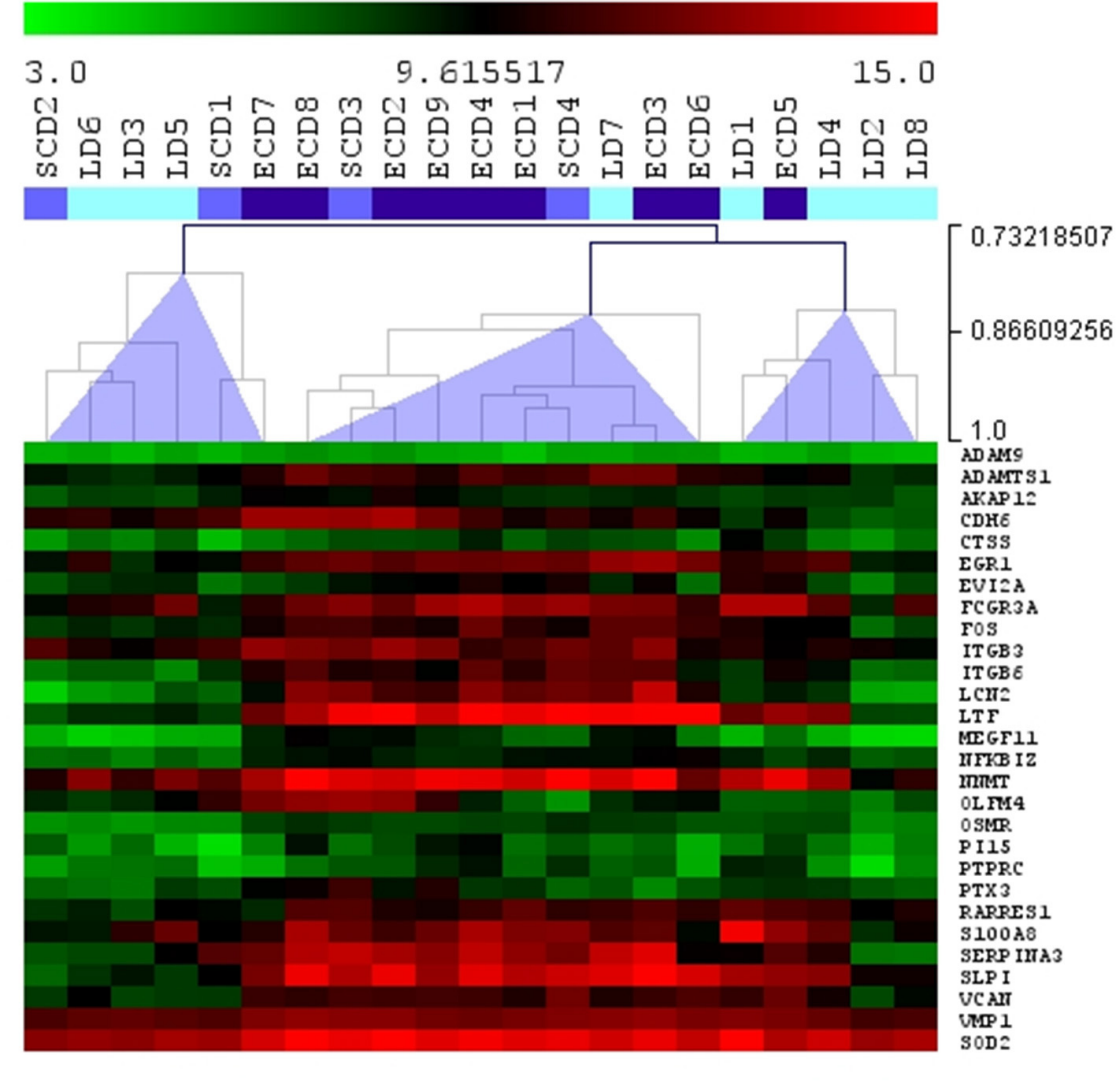
Hierarchical clustering (Spearman rank correlation) for 28 injury-repaired associated transcripts related to acute kidney injury measured in early indication biopsies with borderline changes in different donor categories using Agilent microarray. Light blue: LD, living donor; intermediate blue: SCD, standard criteria donor; dark blue: ECD, expanded criteria donor. Most acute kidney injury transcripts were increased in the second cluster, formed in 70% by grafts from ECD donors.

From 30 injury-repaired associated transcripts related to acute kidney injury (AKI) described by Famulski et al. (12), 28 were measured on the chip in our study, and 19 (64%) of those transcripts were significantly up-regulated in ECD compared to LD donors (Figure 3). Five of those transcripts, *LCN2*, lipocalin 2; *LTF*, lactotransferrin; *VCAN*, versican; *ITGB6*, integrin beta 6; and *SERPINA3*, serpin peptidase inhibitor, were among top ranked significant transcripts that differentiated ECD from LD. Of note, three of 19 AKI transcripts (*LTF, LCN2*, and *SERPINA3*) were more than 10 times more regulated in ECD as compared to LDs (fold change >10).

## Discussion

Donor kidney quality significantly affects kidney transplantation outcomes. It is widely accepted that transplantation of kidneys from elderly marginal donors results in inferior renal function and limited graft life. Expanded criteria donor kidney graft recipients typically suffer from more frequent DGF and acute rejection. In this study, however, the DGF rate was similar between ECD and SCD cohort but much higher than in the LD group (37, 32, and 6%, respectively). Similarly, higher DGF rate was found in respective groups when analyzing a larger cohort of 254 late biopsies (22% in SCD, 18% in ECD, and 4.5% in LD), which may reflect different therapy used in the ECD group. A recent multicenter study reported DGF frequency to be 2.24× higher in ECD compared to SCD recipients (*P* = 0.02) (13). The reason seems to be associated with aggravated alloimmune response and fibrogenesis in already-injured organs. Apart from conventional histological assessment, little is known about molecular pathways typical of marginal kidney grafts. Increasing knowledge in this area may lead to improvements in predicting premature graft loss and adapting therapy appropriately.

To the best of our knowledge, our study is the first to compare intragraft transcriptional profiles from different donor categories in the early posttransplant period, in fact within first 14 posttransplant days. Previous studies have compared preimplant donor biopsies (14) and 0 h (4, 15–17) or postreperfusion (18) graft biopsies from different donor categories. Because all of the indication biopsies we analyzed were diagnosed as borderline changes with no previous pathology, modifications in the transcriptome among donor categories could not have been influenced by different underlying pathological processes. Using microarray, we found higher expression of inflammation- and extracellular matrix remodeling–associated transcripts in the kidney allografts of ECD donors compared with other donor categories.

Compared to the ideal LD group, we observed increased transcripts associated with inflammatory, wounding, and defense responses; complement and coagulation cascades; and cytokine–cytokine receptor interaction pathways in the ECD cohort. This observation seems to be in line with a study by Mueller describing up-regulation of acute phase proteins, complement components, and chemokines in postreperfusion implant biopsies obtained from deceased (compared to living) donors (4). Collectively, this suggests that early transcriptional activations persist at least up to 14 days posttransplant, during which time the biopsies in our study were performed.

In our study, ECD-derived biopsies exhibited increases in several transcripts associated with extracellular matrix remodeling such as thrombospondin 2 (*THBS2*), collagens (*COL6A3, COL1A1*), synaptic vesicle glycoprotein (*SV2B*), and interleukin 11 (*IL11*). Increased expression of *THBS2*, which plays a role in extracellular matrix remodeling, was previously detected in kidney allografts suffering from acute rejection (19). The dominant profibrotic role of *IL11* in the heart and kidneys was recently described (20). An experimental study found increased expression of *ANGPTL4* to be an early biomarker of podocyte injury in a minimal change disease rat model (21). It's up-regulation preceded heavy proteinuria and increased urinary ANGPTL4 protein levels.

Next, in our study, proline-rich 35 (*PRR35*) and *FGF9* were significantly down-regulated in biopsies from ECD donors compared to other cohorts. In another study, expression of FGF9 in biopsies with AKI was lower than in biopsies with primary graft function (22).

Interestingly, *PRR35* and *CD163L* were the most significantly deregulated genes in ECD and LD cohorts, with *PRR35* gene transcripts nearly three times lower in biopsies from ECD donors compared to LD. PRR35 is a protein-coding gene of unknown function. *CD163L*, a macrophage scavenger receptor associated with the anti-inflammatory response and tissue remodeling, has been shown to exhibit three times higher expression in ECD donors (23).

Most importantly, we found significant expression of AKI-related transcripts in ECD kidney grafts. This information is in line with previous “0 h” biopsies study (17). Thus, higher AKI transcripts reflect parenchymal injury associated with donor age and ischemia time and sustain at least 14 days after transplantation, the most critical time period for generation of initial alloimmune response.

In our study, patients with ECD grafts experienced worse renal function at 3 years (median eGFR, 0.65 mL/s) compared to SCD (median eGFR, 0.85 mL/s) and LD grafts (median, 0.94 mL/s). This suggests a higher risk of premature graft loss, although in our study we found no differences in 5 year graft survival between donor categories, which is perhaps unsurprising given the inconclusive results of other studies (2, 7, 24–26). Although the effect of marginal kidneys on graft outcomes has been previously described, it has not been evaluated in a well-defined cohort of patients with the same first pathology of “mild rejection” during the early posttransplant period.

In our early biopsies of ECD patients with borderline changes, the transmission of chronic histological changes was more common, represented by vascular intimal fibrosis (cv), arteriolar hyalinosis (ah), and tubular atrophy (ct) Banff scores compared to biopsies of both SCD and LD categories. The association of higher chronic histopathological Banff scores in biopsies from marginal donors with graft dysfunction or DGF has been reported by other studies (6, 27, 28). In our study, we did not find significantly higher interstitial fibrosis (ci) scores or higher expression of fibroblast-associated transcripts in early BL biopsies of ECD individuals compared to other donor categories. This corresponds to the results of a recent study where indication biopsies performed early after transplantation had higher expression of AKI-associated transcripts than fibroblast-associated transcripts (29).

The sample size for our analysis of graft function and survival (*n* = 269) among particular categories was satisfactorily large. Nevertheless, microarray transcriptome analysis was performed only in a small subgroup (*n* = 21) of patients, representing a possible limitation of our study. However, the main conclusion drawn from our transcriptome analysis is higher activation of immunity, inflammation, and extracellular matrix remodeling in biopsies from marginal donors seen even within 14 days post-transplant. Additionally, because of low number of differentially expressed genes after correction for multiple testing, the unadjusted *p* cutoff < 0.05 and fold change >2 were used instead in statistical analysis. Nevertheless, the high overlap of increased transcripts in marginal donors with the already described molecular AKI injury (12)–related transcript set supports our results of gene annotation analysis. The aim of our study was not to search for any biomarkers requiring larger sample size and validation, but to examine the main transcriptional pathways activated in marginal donors in the early posttransplant period.

In our study, the early borderline changes in ECD kidneys were characterized by the most increased regulation of inflammation, extracellular matrix remodeling, and AKI transcripts in comparison with SCD and LD grafts, respectively. It is likely that ECD-related transcripts were boosted by already present vascular changes in comparison with SCD kidneys and similarly in SCD kidneys by longer ischemia in comparison with LD kidneys. Therefore, chronic vascular changes and cold ischemia time aggravate inflammation and thus contribute to worse outcomes of these grafts. Our data are therefore in line with current praxis where ECD kidney recipients often receive T cell–depletive induction therapy.

## Data Availability Statement

The datasets generated for this study can be found in the Gene Expression Omnibus database under ID GSE134386.

## Ethics Statement

The study was approved by Ethics Committee of the Institute for Clinical and Experimental Medicine and Thomayer Hospital with Multi-Centre Competence under number G-16-06-09. The patients/participants provided their written informed consent to participate in this study.

## Author Contributions

PH and OV designed and wrote the manuscript. PH, ZK, MD, JS, JM, and EH performed the research. VS, JK, and PH participated in the data analysis. OV supervised the research.

### Conflict of Interest

The authors declare that the research was conducted in the absence of any commercial or financial relationships that could be construed as a potential conflict of interest.

## Abbreviations

AKI: Acute kidney injury
CI: Confidence interval
DGF: Delayed graft function
ECD: Expanded criteria donor
eGFR: Estimated glomerular filtration rate
HLA: Human leukocyte antigen
LD: Living donor
rATG: Rabbit polyclonal antithymocyte globulin
PRA: Panel-reactive antibody
SCD: Standard criteria donor
HR: Hazard ratio.
