# Supplementary material for: Molecular Fingerprints of Borderline Changes in Kidney Allografts Are Influenced by Donor Category
Source: Front Immunol. 2020 Mar 25;11:423. doi: 10.3389/fimmu.2020.00423 (PMC7109293; doi:10.3389/fimmu.2020.00423)
Supplement: Supplementary file 1 [file Data_Sheet_1.pdf]

Supplementary Material

1 Supplementary Figures and Tables

Supplemental Table 1. Demographic of patients with early BL in subgroups of SCD, ECD and living donors.

	**SCD** (n=109)	**ECD** (n=109)	**LD** (n=51)	P value* ECD vs SCD vs LD	P value# ECD vs SCD
Donor age, years	40 [4,75]	61 [4,79]	51 [30,76]	**<0.001**	**<0.001**
Recipient age, years	50 [20,80]	55 [28,75]	42 [19,67]	**<0.001**	**0.029**
Peak PRA	4 [0,89]	4 [0,100]	0 [0,46]	**<0.001**	0.596
HLA mismatch	4 [0,6]	3 [0,6]	3 [0,6]	0.313	0.127
Cold ischemia, hours	15.4 [0,25]	16.9[0,27]	0.8 [0,24]	**<0.001**	**0.020**
Dialysis vintage, months	23.4 [0,110]	22[1,128.4]	3.5 [0,144]	**<0.001**	0.945
DGF, n (%)	35 (31.8%)	40 (36.7%)	3 (5.9%)	**<0.001**	0.479
Recipient sex, male, n (%)	76 (69.7%)	83 (76.1%)	42 (82.4%)	0.209	0.361
Retransplantation, n (%)	14 (12.7%)	11 (10.1%)	0	**0.027**	0.164
Donor sex, male, n (%)	56 (45.2%)	58 (46.8%)	10 (8.1%)	**<0.001**	0.893

*Differences among groups were calculated by Kruskal-Wallis test.
Differences among groups were calculated by Mann/Whitney test.

Supplemental Table 2. Histological findings of biopsies with early BL in subgroups of SCD, ECD and LD analyzed by microarray.

**Banff score**	**SCD (n=4)**	**ECD (n=9)**	**LD (n=8)**	**P value (ANOVA)**
Glomerulitis (g)	0±0	0.22±0.44	0.13±0.35	0.620
Chronic glomerulopathy (cg)	0±0	0±0	0±0	1
Interstitial inflammation (i)	0.5±0.58	0.67±0.5	0.63±0.74	0.904
Tubulitis (t)	1.0±0	1.23±0.67	1.5±0.93	0.515
Total inflammation (ti)	0.75±0.5	0.67±0.8	0.5±0.76	0.767
Tubular atrophy (ct/TA)	0.5±0.58	0.89±0.6	0.75±0.46	0.528
Interstitial fibrosis (ci/IF)	0.25±0.5	0.56±0.73	0.63±0.52	0.607
Vascular intimal fibrosis (cv)	0.75±0.5	1.61±0.93	0.63±0.52	**0.028**
Arteriolar hyalinosis (ah)	1.25±1.2	1.31±1.11	0.88±0.99	0.699
Arteriolar hyaline thickening (aah)	0.5±1	0.44±1	0.25±0.7	0.870
Peritubular capillaritis (ptc)	0±0	0.22±0.67	0±0	0.537

1.1 Supplementary Figures

Supplemental Figure 1. Kidney graft function during 3 years follow-up after indication biopsy stratified by donor category. SCD vs LD, $p=0.48$. SCD vs ECD, $p<0.001$, LD vs ECD, $p<0.001$. Differences in kidney graft function among SCD, ECD and LD were calculated by GLM repeated model.

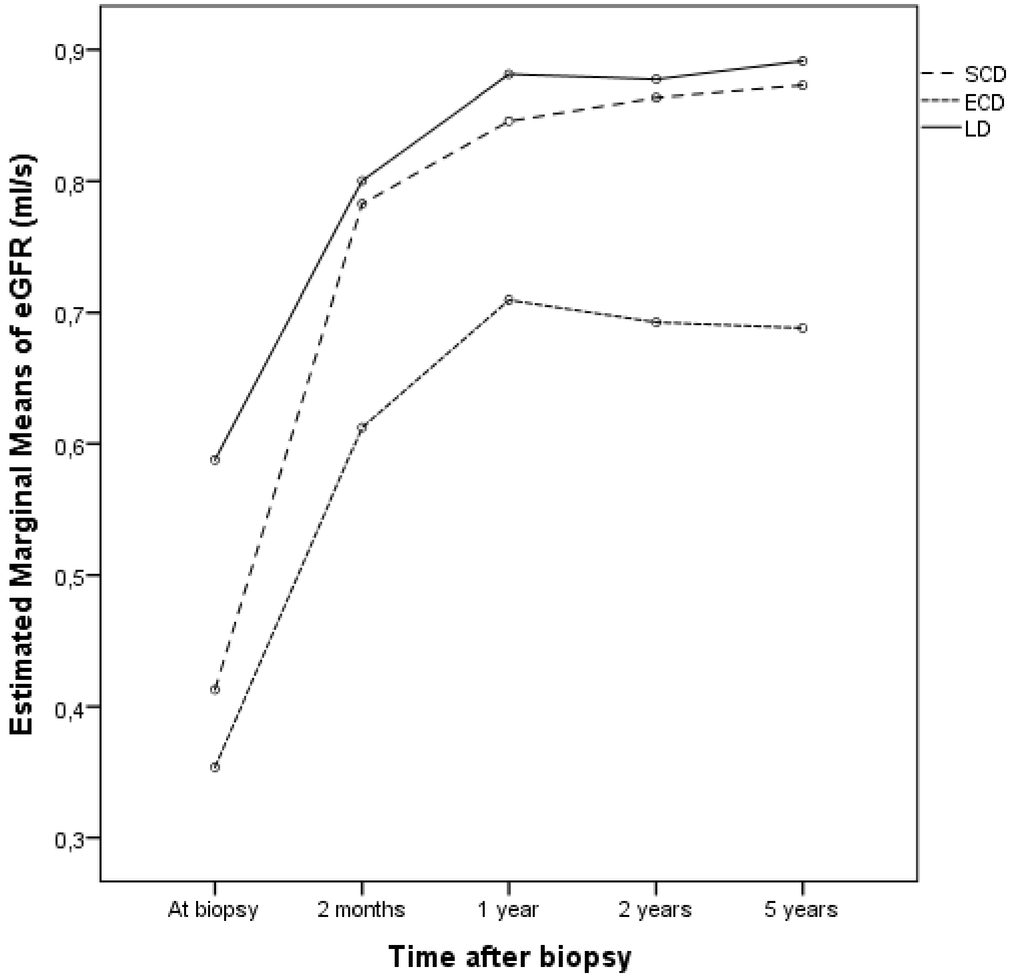

Supplemental Figure 2. Five-year graft survival (A.) and Rejection-free interval (B.) of patients with early borderline changes according to donor category.

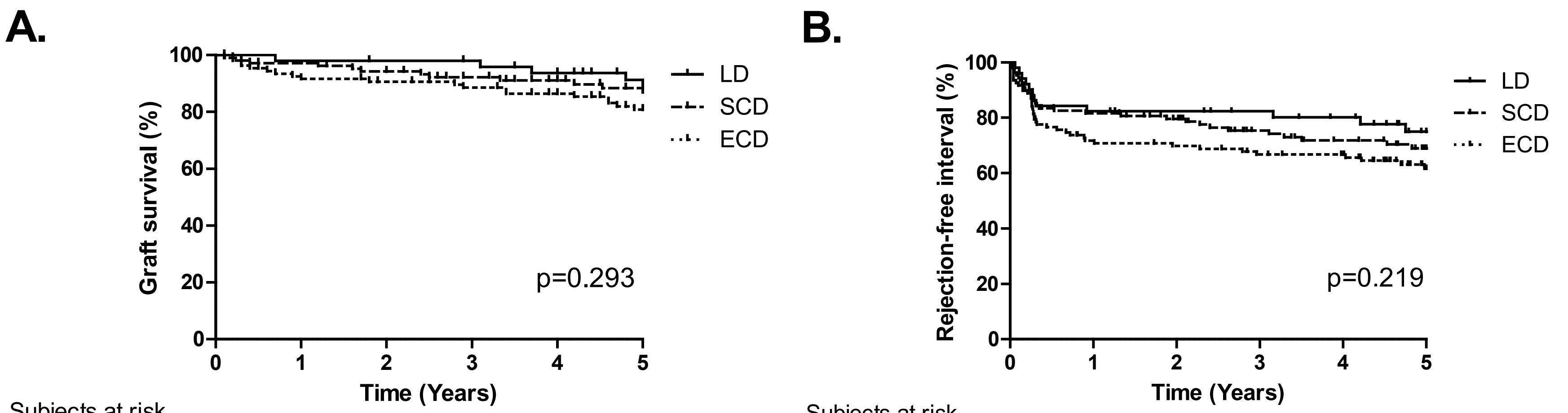

Subjects at risk						
LD	51	51	50	48	43	37
SCD	109	102	97	82	71	63
ECD	109	98	95	87	80	68

Subjects at risk						
LD	51	43	41	38	35	24
SCD	109	88	80	65	56	46
ECD	109	74	71	66	63	40
